# A three-dimensional model of terrain-induced updrafts for movement ecology studies

**DOI:** 10.1186/s40462-024-00457-x

**Published:** 2024-03-28

**Authors:** Regis Thedin, David Brandes, Eliot Quon, Rimple Sandhu, Charles Tripp

**Affiliations:** 1 National Renewable Energy Laboratory, Golden, CO USA; 2 Lafayette College, Easton, PA USA

**Keywords:** Complex terrain, Energy landscape, Large-eddy simulation, Movement ecology, Orographic soaring, Orographic updraft modeling, Soaring birds

## Abstract

**Background:**

Spatially explicit simulation models of animal movements through the atmosphere necessarily require a representation of the spatial and temporal variation of atmospheric conditions. In particular, for movements of soaring birds that rely extensively on vertical updrafts to avoid flapping flight, accurate and reliable estimation of the vertical component of wind is critical. The interaction between wind and complex terrain shapes both the horizontal and vertical wind fields, highlighting the need to model the coupling between local terrain features and atmospheric conditions at scales relevant to animal movement.

**Methods:**

In this work, we propose a new empirical model for estimating the orographic updraft field. The model is developed using computational fluid dynamics simulations of canonical atmospheric conditions over moderately complex terrain. To isolate buoyancy and thermal effects, and focus on terrain-induced effects, we use only simulations of a neutrally stratified atmosphere to develop the model. The model, which we name Engineering Vertical Velocity Estimator (EVVE), is simple to implement and is a function of the underlying terrain elevation map, the desired height above ground level (AGL), and wind conditions at a reference height (80 m). We validate the model with data from the Alaiz mountain (Spain) field campaign.

**Results:**

Compared to observations, the proposed improved model estimates the updrafts at 120 m AGL with a mean error of 0.11 m/s ($\sigma =0.28$ m/s), compared to 0.85 m/s ($\sigma =0.58$ m/s) for its baseline. For typical land-based wind turbine hub heights of 80 m AGL, the proposed model has a mean error of 0.04 m/s ($\sigma =0.25$ m/s), compared to baseline 0.54 m/s ($\sigma =0.45$ m/s) estimations. We illustrate an application of the model in movement ecology by comparing simulated tracks and presence maps of golden eagles (*Aquila chrysaetos*) moving across two distinct landscapes. The tracks and presence maps are obtained using a simple heuristic-based movement model, with the updraft field given by the proposed model and a wind vector-based estimation approach that is currently in wide use in movement ecology studies of raptors and other soaring birds.

**Conclusions:**

We highlight that movement model results can be sensitive to the underlying orographic updraft model, especially in studies of fine-scale movements in regions of complex topography. We suggest adopting the proposed model rather than the wind vector estimation method for studies of soaring bird movements.

## Introduction

The movements of birds in flight are closely linked to the motions of their surrounding aerial habitat, i.e., the atmosphere [12, 28, 32, 34, 43, 55, 58]. Thus, a quantitative understanding of spatio-temporally varying wind flows in the lower portion of the atmospheric boundary layer are critically important elements in understanding movement patterns from scales ranging from tens of meters to thousands of kilometers. Recent advances in tracking devices have provided a highly detailed picture of animal movement patterns in the heterogeneous aerial environment. One particularly useful concept that has emerged from coupling animal tracking data with concurrent environmental data during movements in fluid flows (aerial and marine) is that of the energy landscape [57, 67]. According to Shepard et al. [57], energy landscapes represent the energetic cost of transport and can be used to indicate whether—and to what extent—movement patterns are influenced by environmental conditions that support or hinder movement. Although the energy landscape is likely not the sole determinant of movement decisions, the concept is highly relevant for large soaring birds (condors, vultures, eagles, storks, pelicans) for which powered flight is energetically costly [43]. Thus, the spatial and temporal availability of updrafts (vertical air motion) as well as the horizontal wind components are important determinants of movement patterns, flight altitudes, and velocities [6, 17, 26, 31, 33, 36, 46, 56, 66].

For soaring species, the modeled vertical air velocity field has been used as a proxy for the energy landscape. Shepard et al. [57] include examples (see their Figs. 3 and 4) for two species of large soaring birds, Andean condor (*Vultur gryphus*) and black vulture (*Coragyps atratus*). In both cases, energy landscapes were constructed as simulated mean vertical air velocity over complex terrain using a numerical reanalysis model [63]. Bohrer et al. [6] developed continental scale maps of thermal and orographic updraft potential using simplified estimation methods (now incorporated into the Env-DATA track annotation system in Movebank at www.movebank.org) for characterizing the use of thermal and orographic updrafts by turkey vultures (*Cathartes aura*) and golden eagles (*Aquila chrysaetos*) during long-distance migration in North America. Santos et al. [51] showed good correlation of flight modes of migrating black kites (*Milvus migrans*) with the spatial distribution of updrafts modeled at 30–100 m resolution using a modification of Bohrer et al. [6] with land surface temperature data. Hanssen et al. [21] used this same approach to map updrafts for selected dates at 10–100 m resolution on the 680 km^2^ island of Hitra, Norway. The results showed that the presence of white-tailed eagles (*aliaeetus albicilla*) flying at low altitude was positively correlated with orographic updraft intensity but negatively correlated with thermal updraft intensity [21]. In a study of the migratory movements of white storks (*Ciconia ciconia*), Scacco et al. [54] found that static features of the landscape (elevation, topographic roughness, normalized difference vegetation index) were effective in identifying areas of uplift but not intensity of uplift. In earlier work in which a fluid-flow model was applied to simulate golden eagle migration patterns over complex terrain [7], the term “conductivity field” was used rather than energy landscape to describe the modeled updraft field; however, the concept is similar, with high conductivity being equivalent to high available energy for subsidizing movement.

Although some movement ecology studies have used computational fluid dynamics-based models to simulate vertical flow velocities over complex terrain (e.g., [53, 57]), as spatial scale increases such approaches are often prohibitively expensive and time-consuming. Accurate estimation methods of such vertical flows are needed for understanding fine-scale animal movement patterns as well as the potential impacts of wind energy developments on soaring birds in areas of complex terrain. There is keen interest in developing reliable methods for siting wind facilities in areas of lowered risk [1, 21, 40, 42]. In particular, studies have demonstrated that low-altitude flight of several different raptor species is linked to terrain slopes and use of orographic updrafts rather than soaring and gliding in thermals, which typically occurs at much higher altitudes [2, 21, 25, 44, 46]. Flight within the “rotor-swept zone” of land-based turbines (approximately 30 to 150 meters, depending on the turbine model) is a particular focus of our work, and accurate models of orographic lift over complex terrain are central to developing turbine collision risk models that account for flight behavior of soaring birds. There are also potential engineering applications of improved orographic updraft models in the flight of unmanned aerial vehicles and autonomous gliding vehicles [5, 8, 19, 30, 41]. Studies of such flows have been performed using idealized hills [4, 61], but real, complex terrain may include the coupled effects of several nearby features that modify the wind field through channeling or sheltering effects. For instance, speedups at instrumented locations near the Altamont Pass wind farm in California were reported to be affected by upstream terrain features [65]. The same work noted that the speedups observed are more complex than those predicted by previous studies.

Many studies in the movement ecology literature (e.g., [11, 21, 40, 49, 51, 55]) have used a wind vector-based estimation model for orographic updraft velocity [6, 7] that is based on a digital elevation model (DEM) for computing local terrain slope and aspect coupled with a known horizontal wind speed and direction. The model is given by a nondimensional updraft coefficient

1 $$\begin{aligned} W_0 = \sin \theta \cdot \cos \left( \alpha -\beta \right) \end{aligned}$$

where the first term is a measure of the local slope angle $\theta$, and the second term is the cosine of the angle between the the wind direction $\alpha$ and the terrain aspect $\beta$. The updraft coefficient value is zero in flat regions where $\theta =0$. Likewise, the updraft coefficient is the largest when the cosine term is 1, which happens when the wind direction is orthogonal to the direction of slope. For winds parallel to slopes the cosine term is zero, and thus no updraft is present. The model, hereafter referred to as $\textsc {BO04}$, does not explicitly state the multiplying quantity used to dimensionalize the updraft coefficient to an updraft velocity.

Follow-on work by Bohrer et al. [6] introduced $w_0$ as a dimensional updraft potential, being equal to $W_0$ multiplied by the “horizontal ground wind speed.” The imprecise definition of the multiplying velocity $w_0/W_0$ has led to different interpretations and inconsistencies in the application of the model. For example, Hanssen et al. [21] used horizontal wind speed data from a weather station at 13 m elevation, Sage et al. [48] at 2 m, and Dennhardt et al. [11] at 30 m. Santos et al. [51] and Marques et al. [38] also used weather station data, but from an unspecified height. Sandhu et al. [49] used gridded interpolated wind fields at 100 m AGL. Many other authors are not explicit about wind data height used on their study. Furthermore, the $\textsc {BO04}$ model is two-dimensional and is only implicitly dependent on height by means of the multiplying wind speed at the desired height above ground level (AGL). Considering a typical atmospheric boundary layer profile, wind speed increases approximately logarithmically with height AGL in the lowest $\sim$100 m [59], whereas the effect of terrain on the flow field declines with height AGL, extending only a few hundred meters above the ground surface [28, 61]. Also note that the updraft coefficient is a measure of the terrain properties at the point directly below the location of interest, rather than the region surrounding or upstream/downstream of that point. Thus, in practice, the $\textsc {BO04}$ model results in an estimated updraft field that is sensitive to local, rapid changes in slope and/or aspect, which is an inherent characteristic of high-resolution DEMs. The irregular, rapid changes of these terrain-based quantities can then result in local overestimation or underestimation of the actual updraft field, as will be shown later.

Despite its extensive use in the movement ecology literature, $\textsc {BO04}$ is a highly simplified representation of a complex process and has not been compared to numerical flow simulations nor validated with field measurements. As a two-dimensional model, it has limited value in providing understanding or prediction of three-dimensional flight patterns of flying animals. In this work, our objective is to build upon the $\textsc {BO04}$ updraft estimation model and propose empirical modifications based on large-eddy simulation (LES) modeling of flow over complex terrain. We only consider orographic effects under conditions of neutral atmospheric stability—that is, we do not include buoyancy effects due to variation of potential temperature that might accompany terrain-induced updrafts in the field. We also focus solely on the updraft field on the windward side of terrain, as the leeward side is subject to flow separation effects and more complex turbulent flows than a simplified model is capable of capturing. The goal is an improved estimation approach that provides more realistic matches to LES simulations and to field data, but retains computational efficiency for spatially explicit flow modeling at scales relevant to movement ecology studies.

We note that the model developed in this work is available to the public on GitHub, under the project “Engineering Vertical Velocity Estimator” [62]. Interactive examples are provided in accompanying Jupyter notebooks in the repository.

## Methods

In this section, we describe the methodology behind each of the steps used to develop, validate, and apply the improved updraft model. Specifically, “Large-eddy simulation methodology” section describes the LES model and details used to obtain the terrain-resolving turbulent flow field. Next, in “Model development” section we describe the updraft model development, separated in two parts. First, in “Parametric study using synthetic terrains” section, we perform a parametric study using simple gaussian hill geometries to obtain a height adjustment $f_h$ based on the wind speed at a reference height $V_\text {ref.}$. In "Model tuning with real terrains" we use LES solutions over real terrains of different levels of complexity to further tune the model and develop adjusting factors based on a measure of terrain channeling and sheltering effects $f_{Sx}$, and terrain complexity $f_{tc}$. In “Model validation with field observations” section, we describe model validation using field measurements of vertical velocities over complex terrain. Finally, in “Model illustration with movement model simulations” section we describe the application of a movement model for golden eagles, using both the original and the adjusted model.

### Large-eddy simulation methodology

We use the Simulator for Wind Farm Application (SOWFA), developed at the National Renewable Energy Laboratory. SOWFA is an open-source turbulence-resolving LES tool, focused on wind energy applications. Some of the capabilities of SOWFA are its ability to represent full diurnal cycles driven by large-scale mesoscale conditions, different canonical stability states, realistic atmospheric features (e.g., low-level jets, weather fronts, or gravity waves), and features like wind turbines and complex terrain. SOWFA has been extensively used and validated in works within the wind energy community [9, 13, 14, 20, 24, 27, 39]. Its capability of including complex terrain and capturing terrain-generated turbulence has also been investigated [22, 47]. An LES approach is used in SOWFA, meaning that turbulence is resolved in the computational fluid dynamics simulation down to the grid scale, and smaller, sub-grid-scale turbulence is modeled. The reader is referred to the work of Churchfield et al. [10] for more details on SOWFA. The effect of the rough planetary surface is modeled by the Schumann surface stress boundary condition, and the aerodynamic surface roughness is kept constant at 0.15 m.

In the numerical studies conducted, we take a two-step approach in coupling complex terrain to atmospheric turbulence. First, we execute a case where the computational domain has periodic boundary conditions on its lateral boundaries and a flat bottom boundary. This step is known in the literature as a precursor simulation. The goal of the precursor is to spin up the background atmospheric turbulence. When turbulence is developed—identified by the convergence of profiles of turbulence metrics and a resolved energy cascade that follows Kolmogorov’s law [29]—information at the lateral boundary planes is saved. In this step, we assume horizontally homogeneous turbulence by the use of cyclic boundary conditions on the lateral boundaries. Next, a second simulation is set up where the bottom boundary is conformed to a digital elevation model of the terrain geometry and the boundary data saved from the precursor are used as boundary conditions at the inflow planes. The real terrains considered in this work are obtained using NASA’s Shuttle Radar Topography Mission [64] digital elevation model, with resolution of approximately 30 m.

The domain size and grid resolution are responsible for the upper and lower bounds of the scales captured. Due to horizontal homogeneity, the largest scales are of the order of the domain size. The lowest scales resolved are of the order of 4–5 times the grid size [45]. Simulation of atmospheric boundary layers is a multiscale problem and can quickly become computationally expensive if a wide range of scales is needed. Here, we select domain sizes, resolution, and simulation time such that the temporal and spatial scales of interest are properly captured. Note that throughout this work, the LES results are presented in a time-averaged fashion.

### Model development

#### Parametric study using synthetic terrains

**Fig. 1:**
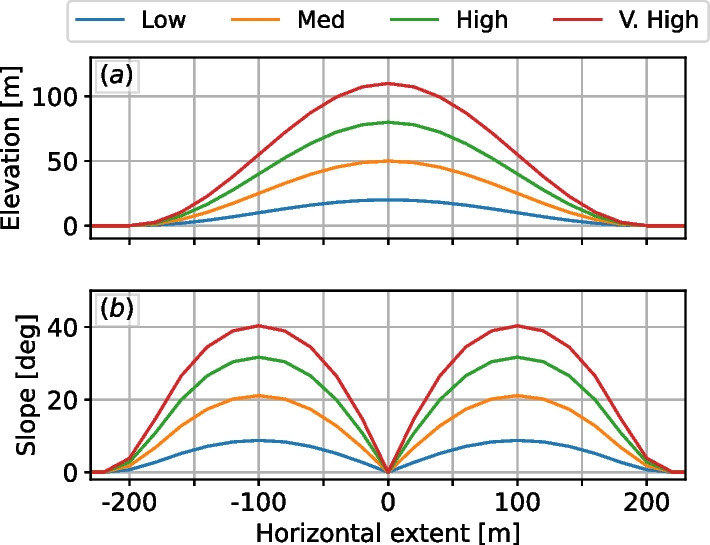
The four Gaussian hill geometries used to perform the parametric study. **a** Elevation; **b** orographic slope

We developed the empirical model using a parametric study of idealized Gaussian hill geometries. The conditions are canonical neutrally stratified boundary layers, with a wind speed of 8 m/s at a reference height of 80 m AGL. This reference height was used throughout this study because of the availability of measurements from reanalysis datasets at this height and because it is a typical measurement height on tall meteorological masts where horizontal wind speed data are often collected for wind-energy applications. The numerical setup includes a $3\times 3\times 1$ km domain with uniform 10-m grid resolution. A capping inversion is enforced by a 10 K stable stratification between 750 and 850 m AGL, effectively limiting the growth of the boundary layer. The domain extents and resolution used are typical values from the atmospheric and wind energy LES community (see [9, 14, 39] and references therein). In this parametric study, we analyze four hills with varying steepness levels, shown in Fig. 1. We investigate the effect of the wind angle $\alpha -\beta$ by analyzing two angles, 0 and 45 degrees. In the 0 degrees case, hereafter called orthogonal flow, the winds are orthogonal with the ridgetop and, by Eq. (1) the resulting updrafts are the largest possible. In 45 degrees flow, hereafter called oblique flow, the winds approach the slopes at a 45 degree angle. In this study, the hills were designed so that the two of higher steepness are expected to produce separated flow under an 8 m/s wind. The flow separation in such cases induces significantly higher turbulence levels.

**Fig. 2:**
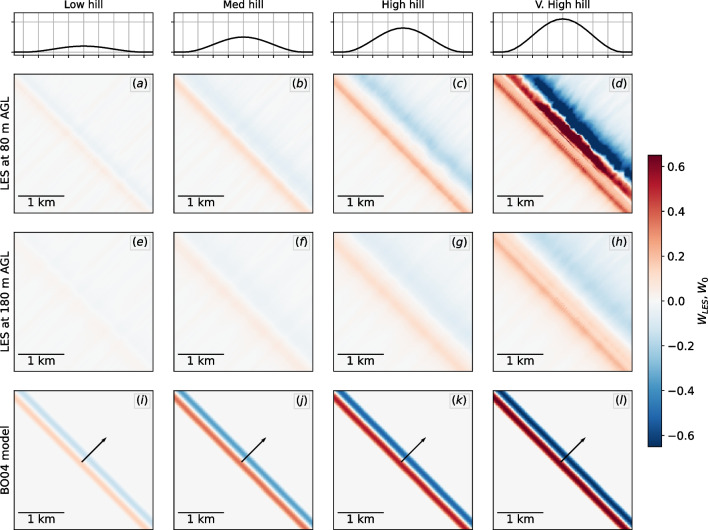
Large-eddy simulation solutions of orthogonal flow compared to the $\textsc {BO04}$ model around hills placed diagonally, with the hilltop placed exactly on the diagonal of the domain shown. Each row corresponds to a height AGL, as indicated by the labels on the left. The black arrow at the bottom row represents the direction of the flow. Each column corresponds to a hill, reproduced at the top row (to scale). Nondimensional vertical velocity from **a**–**d** LES at 80 m, **e**–**h** LES at 180 m, and **i**–**l** $\textsc {BO04}$ model. Note that $\textsc {BO04}$ model is height-independent

In order to keep the same incoming flow to both the orthogonal and oblique cases, instead of executing two different wind directions, we opt to manipulate the geometry. Our background wind field was simulated for incoming southwest winds. That means that for our oblique case, the hills shown in Fig. 1 ran from north to south; whereas for the orthogonal flow case, the hills were placed diagonally on the domain, running from the northwest to the southeast. Figure 2 shows the nondimensional time-averaged updraft field obtained from LES at selected heights and estimations from the $\textsc {BO04}$ model for the orthogonal flow case. In the figure, the top of the hill is exactly on the diagonal of the domain (more clearly visible in Fig. 2i–l), and the arrow notes the direction of the wind. It becomes clear that steep hills induce flow separation and high values of vertical velocity. The imprint of the hill is less evident as the height increases; that is, the updraft field becomes less strong as the height AGL increases, as expected.

This first study also reveals that positive updraft can be observed on the leeward side of the hill. Asymmetry in the windward and leeward sides of hills is impossible to be represented by the $\textsc {BO04}$ model. Even with LES, the turbulent flow on the leeward side of steep terrain features can be challenging to model. In the present study, this secondary updraft zone has not been considered.

**Fig. 3:**
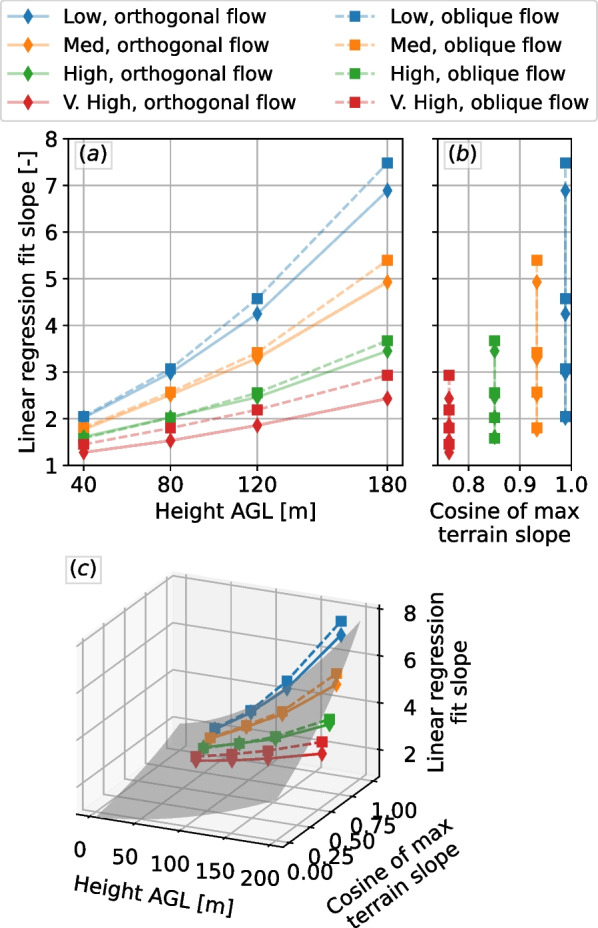
Slopes of the linear fit on the relationship of the positive vertical velocity for each of the hills and flow direction investigated. Panels **a** and **b** show different views of the 3-D data shown in **c**. The gray surface in **c** is the surface given by Eq. 2

Analogous LES results to those presented in Fig. 2 were developed for selected AGL values, roughly covering a typical land-based wind turbine rotor-swept zone: 40, 80, 120, and 180 m AGL. For each scenario at each height, the $\textsc {BO04}$ model results are compared with the LES on a scatter plot. A clear linear relationship is observed for the two less-steep geometries, whereas no clear relationship is found for the steeper hills. We found that the points not following a linear relationship in those cases are related to the separated flow region and strong turbulence. When we consider only the positive vertical velocity values, however, the linear relationship is recovered for all scenarios. Therefore, for each combination of flow angle, hill steepness, and height, we obtain the slope of the linear regression fit of the scatter data. The linear regression fit slope related to each case is shown in Fig. 3. The variation of the slope versus both the height AGL and the cosine of the maximum orographic slope value (see Fig. 1) is presented. Considering that at the limit, a flat terrain produces no orographic-induced vertical velocity, the variation of the slope of the linear regression fit with respect to height can be approximated by a second-order polynomial, while its variation with the cosine of maximum hill slope can be approximated by an exponential relationship.

We fit these points shown in Fig. 3 with a surface that varies quadratically with the height *h*, and exponentially with the cosine of the slope $\theta$, in an expression of the form

2 $$\begin{aligned} f_h = \left( ah^2+ bh + c \right) \cdot \left( d^{-\cos \theta + e} \right) + f \end{aligned}$$

where $f_h$ is an adjustment factor to be used on top of the $\textsc {BO04}$ model. We approximate the surface fit, which yields $a=0.00004$ m^-2^, $b=0.0028$ m^-1^, $c=0.8$, $d=0.35$, $e=0.095$, and $f=-0.09$. While this adjustment is mathematically valid for all heights, given the fit is performed at heights within the rotor-swept zone, care should be taken when using it for heights much lower than 30 m and much higher than about 200 m. An illustration of the fitted surface is given in Fig. 3c.

**Fig. 4:**
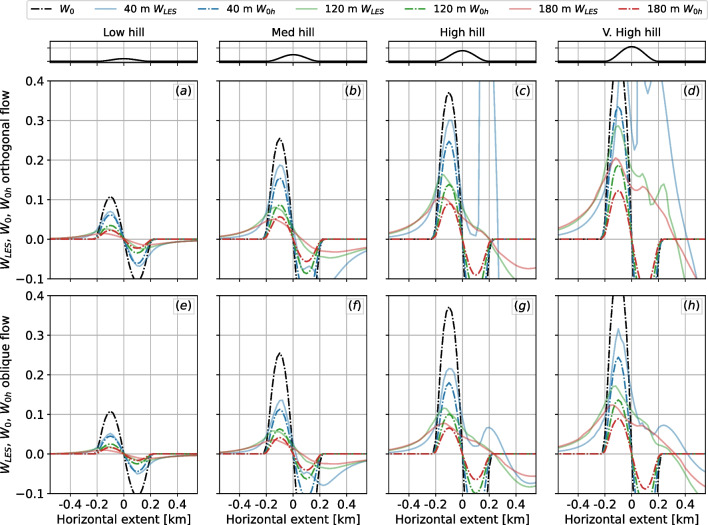
Curves of nondimensional updraft velocity averaged along the ridge line: **a**–**d** orthogonal flow; **e**–**h** oblique flow. While the height adjustment $W_{0h} = W_0 / f_h$ improves the magnitude with respect to the LES solutions, it does not account for nonzero updrafts upstream of the hill

A straightforward way to visualize the adjusted results is to look at their cross sections as opposed to the whole domain as shown previously in Fig. 2. The cases investigated, even though they are fully three-dimensional, can be averaged in the ridge-wise direction and presented as two-dimensional profiles, shown in Fig. 4 for selected heights. The figure compares LES results with the height-adjusted model (Eq. 2) and the original model. Naturally, the height-adjusted results will be a better match on these results since the correction was developed with the same data. It is of interest, however, to note how nonzero updraft regions can extend far upstream of the geometry on the LES results. The extent length seems to be related to the steepness of the hill, where steeper hills have updraft regions extending further upstream.

Taking a closer look at the upstream extent up vertical velocities in each case, we can see that the updraft is not only a function of the slope present directly below the point considered but also contains information from surrounding orographic features. At 40 m AGL, positive updrafts have been observed between 100 and 250 m upstream from the start of the hill (for the Gaussian geometries under investigation we define the start of the hill as 200 m upstream the hilltop). At higher heights, such as 160 m AGL, such distance can be greater than 600 m. At 160 m, the nondimensional vertical velocity values are less than half of those at 40 m. Therefore, for every location, we need to incorporate information about downstream terrain features. A useful terrain metric in this context is that of wind shelter or exposure. Developed by Winstral et al. [69] and Winstral and Marks [68] with focus on snow accumulation and melt, a terrain exposure/sheltering parameter, *Sx*, was initially formulated based on data from the Green Lakes Valley in Colorado and later validated with data from an exposed site and a sheltered site in the Reynolds Mountain East watershed, located in Idaho. *Sx* is based on the maximum upwind slopes found between the point of interest and a pie-shaped sector extending upwind of the point in the direction of interest:

3 $$\begin{aligned} {\widetilde{Sx}}_{{\widetilde{A}},\text {dmax}} \left( x_i,y_i\right) = \max \left[ \tan \left( \frac{ z\left( x_v,y_v\right) - z\left( x_i,y_i\right) }{\sqrt{\left( x_v-x_i\right) ^2 + \left( y_v-y_i\right) ^2}} \right) \right] \end{aligned}$$

where *z* is the elevation map from a DEM, ${\widetilde{A}}$ is the azimuth of the search direction, $(x_i,y_i)$ are the coordinates of the cell of interest, and $(x_i,y_i)$ are the set of all cell coordinates located along the search vector defined by $(x_i,y_i)$, ${\widetilde{A}}$, and $\text {dmax}$. The extent of the sector ($\text {dmax}$) is a tuning parameter. A negative ${\widetilde{Sx}}$ value indicates an exposed location, whereas a positive value indicates a sheltered location. As recommended by the authors of the model, we average ${\widetilde{Sx}}$ across an upwind window of directions, resulting in a measure that is more robust to both natural and systematic deviations from the recorded data, *Sx*. The mean maximum upwind slope parameter is then given by

4 $$\begin{aligned} Sx_{A,\text {dmax}} \left. \left( x_i,y_i\right) \right| _{A_1}^{A_2} = \frac{1}{n_v} \sum _{A=A_1}^{A_2} {\widetilde{Sx}}_{A,\text {dmax}} \left( x_i,y_i\right) \end{aligned}$$

where $A_1$ and $A_2$ define the outer limits of the upwind window, *A* bisects $A_1$ and $A_2$, and $n_v$ is the number of search vectors in the window defined by $A_1$ and $A_2$.

The *Sx* quantity is used differently in this work. Based on its original definition, it gives information about upstream features, whereas our interest is primarily on downstream features. For that, we flip the wind direction that goes into the *Sx* calculation so that $A = (\text {wdir}+180)\%360$ with wdir being the wind direction given in degrees and the % operator denoting rest of division (modulo operator). The consequence for that is that we have a positive angle on the windward side and negative on the leeward side. We use a 30-degree window for all *Sx* derivations and ${\widetilde{Sx}}$ calculated in 5 degree increments. Given the range of values observed previously, $\text {dmax}$ is selected to be 500 m.

#### Model tuning with real terrains

Next, we develop additional empirical corrections to account for flow effects over real terrain. Real terrain rarely contains isolated features like the idealized hills analyzed above, and thus complex flow patterns are expected to occur, including some wind channeling effects. We emphasize that in the development of the adjustment factors, we do not attempt to model the complex turbulent flow on the leeward side of the terrain; rather, our focus is on the upwind side.

**Fig. 5:**
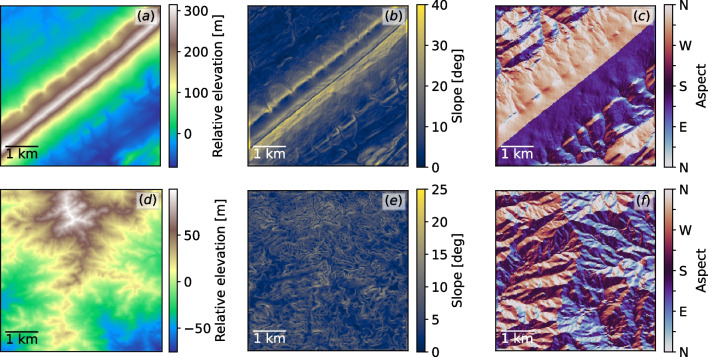
Regions considered for model tuning. **a**, **d** 10 m digital elevation map, **b**, **e** corresponding slope, and **c**, **f** aspect. **a**–**c** Pennsylvania region; **d**–**f** Wyoming region. Note the different scales

We selected two regions containing different types of terrain features, shown in Fig. 5. The first is relatively mildly sloping unstructured terrain near the Top of the World wind farm in Wyoming (referred to as Wyoming region). In this region, flow separation would not be expected except under high wind conditions at a few isolated locations. The second region is within the Valley and Ridge physiography of the Appalachian Mountains in central Pennsylvania, known for its long linear ridges (referred to as Pennsylvania region). The domain in this case includes a steep ridge similar to the idealized one above and represents a more extreme terrain type. In this region, strong separated flow is expected to occur. The choice of these sites is primarily due to the contrast in terrain, but also the Wyoming region has a high density of golden eagles and has had documented fatalities at several wind farms, whereas the Valley and Ridge region of Pennsylvania is a known migratory path for golden eagles.

In this part of the study, the numerical domain extents were increased to $5\times 5\times 2$ km, with a uniform 10 m grid resolution. The taller domain allows the boundary layer to deform according to the underlying terrain without introducing numerical artifacts at upper boundary. The choice of wind speeds and wind directions to be simulated as canonical neutral boundary layers followed typical conditions observed at the site of interest during the years of 2017 and 2018. Historical data were obtained through the Wind Integration National Dataset (WIND) Toolkit [16]. Wind roses of the region reveal that the wind is predominantly from the west during the summer and spring seasons. For the analysis, two wind speeds were chosen. One of 8 m/s representing the low wind speeds observed during the day at those seasons, which corresponds to about 78% of the winds encountered at the site, and another wind speed of 15 m/s, representing an extreme. A wind speed of 15 m/s was only observed during 4% of the time at the region, but represents a challenging, yet realistic scenario that is important to account for in the model.

As mentioned in “Parametric study using synthetic terrains” section, we use the modified *Sx* parameter to account for downstream features. An adjustment for downstream features based on the tangent of the exposure quantity *Sx*, considering the flipped wind direction, is given as

5 $$\begin{aligned} f_{Sx} = 1 + \tan \left[ Sx\left( A=(\text {wdir}+180)\%360, \,\text {dmax}=500 \text { m} \right) \right] \end{aligned}$$

where % denotes the modulo operator, and the wind direction is given using typical wind direction convention where 0 degrees represents wind coming from the north. Note the flipping of wind direction gives us the correct sign of the factor $f_{Sx}$ that allows straightforward multiplication of all the individual factors.

**Fig. 6:**
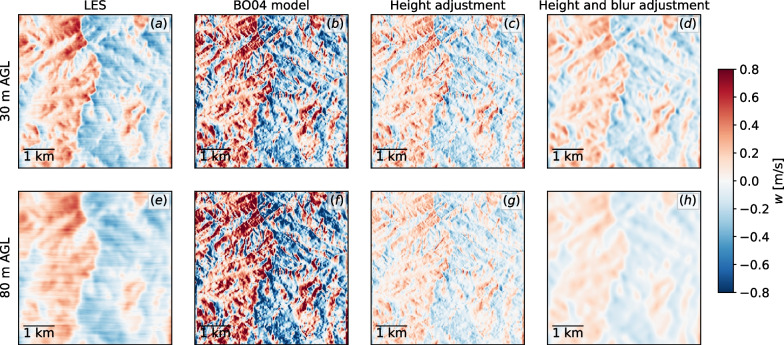
Illustration of the intermediate steps of the model, showing the dimensional vertical velocity field around the Wyoming region (Fig. 5d–f). **a**, **e** LES; **b**, **f** original $\textsc {BO04}$; **c**, **g** proposed model with *Sx* and height adjustment; **d**, **h** proposed model with height and blur adjustment. Each row illustrates one height AGL, indicated on the left

Upon exploration of the LES results, it becomes clear that the impact of small terrain features is less defined at higher heights. That is, the updraft becomes less sensitive to isolated terrain features and instead resembles an integration of the effects of nearby features. Based on observations of the LES results at different heights, we experimented with a direct convolution of terrain metrics with a Gaussian function. Using the slope and aspect quantities blurred by Gaussian kernels resulted in blurred versions of the $\textsc {BO04}$ model updraft field, which more closely matched that obtained using LES. We then devise another aspect of our improved model, which is to use metrics convoluted with Gaussian functions. The original updraft coefficient given by Eq. (1) is then computed using the filtered metrics. The Gaussian blurring kernels have an empirically determined standard deviation $\sigma$ that varies linearly with height in the form of

6 $$\begin{aligned} \sigma = \min (0.8h + 16, \, 300) \end{aligned}$$

where *h* is the height AGL in meters. The minimum guarantees a maximum kernel size at much higher heights. Considering the Wyoming region, the blurring step is illustrated in Fig. 6, showing the dimensional vertical velocity field obtained using LES at different heights, the original model, their counterpart *Sx*- and height-adjusted, and the proposed blurring approach. As noted earlier, the original model is sensitive to small-scale terrain features, resulting not only in localized overestimation and underestimation of the updrafts, but also on a field that is too well defined with respect to the underlying terrain and not realistic.

The blurred adjustment results in a flow field that more closely matches the LES model, although it generally underestimates the values from the LES. Therefore, a final adjustment was developed related to terrain complexity. The Pennsylvania region, with its significant spread in elevation-induced flow separation and high levels of turbulence as well as vertical velocities that exceeded 1.5 m/s at the top of the ridge. The Wyoming region with mildly complex terrain, on the other hand, has mostly attached flows. In order to account for the steep hills where the vertical velocity is significantly higher, we introduce a terrain complexity factor. Many different measures have been used to describe the complexity of peaks, valleys, and ridges—for example, variance of elevations, autocorrelation of elevation, relief, contour density, rugosity, curvature, slope change, aspect change, etc. A good discussion on the topic can be found in the work of Huaxing [23]. For the purposes of this work, we are interested in a metric that is simple and is localized. By localized, we mean that in a large region with heterogeneous terrain, complex regions will be assigned a high value for its local features, while flat regions will be assigned a low value. We derive a simple metric that is based on the mean elevation surrounding each DEM cell and further scaled by the relief in the same local region. A terrain complexity factor is given by

7 $$\begin{aligned} \text {tc} = \frac{ \hspace{0.83328pt}\overline{\hspace{-0.83328pt}\langle z \rangle \hspace{-0.83328pt}}\hspace{0.83328pt}- \langle z \rangle _\text {min} }{ \langle z\rangle _\text {max} - \langle z\rangle _\text {min} } \end{aligned}$$

where the angled bracket symbols refer to quantities within a $500 \times 500$ m area, centered around the cell of interest, and the overbar represents a mean quantity. The expression on the denominator is the relief of each region. The terrain complexity is further multiplied by another parameter, $\Lambda$, which is a linear function of the height AGL, $\Lambda =h/40$. The normalizing value of 40 m was obtained from regression analysis comparing LES data to the model, and represents a mean value across all cases investigated. Finally, the terrain complexity factor is given as the following:

8 $$\begin{aligned} f_{tc} = 1 + \Lambda \cdot \text {tc}. \end{aligned}$$

The $\Lambda$ parameter makes the terrain complexity factor increase with height. It essentially gives a larger weight for high heights without compromising lower heights. This is necessary to counterbalance the second-order polynomial that grows fast as height increases.

Finally, we compound the adjustment factors discussed to come up with an improved model for the orographic updrafts, $w_{0i}$. The final adjusting factor is

9 $$\begin{aligned} F = \frac{f_{Sx} \cdot f_{tc}}{f_h}, \end{aligned}$$

and the model reads

10 $$\begin{aligned} W_{0i} = F \cdot W'_0, \qquad w_{0i} = V_\text {ref.} W_{0i} \end{aligned}$$

where $V_\text {ref.}$ is the horizontal wind speed at a reference height, 80 m, and the $W'_0$ is the original coefficient (Eq. (1)) computed using slope and aspect blurred by the Gaussian kernel with standard deviation given by Eq. (6). As mentioned, the idea is that horizontal wind speeds at this reference height can be obtained either by field experiments (where 80 m is a height where instruments are typically deployed) or by analysis models, thus removing the need to obtain the wind speeds at the height of interest.

Statistical comparison of the resulting model with LES results over the Wyoming and Pennsylvania terrains is included in “Model comparison to LES using real terrains” section, computed using the differences between modeled updraft and LES updraft in regions where the modeled vertical velocities are positive.

To briefly summarize the previous sections, the model development process is comprised of the following steps:

- Replace the multiplying wind speed $w_0/W_0$ of the original $\textsc {BO04}$ model by a reference wind speed obtained at a fixed, reference height, called $V_\text {ref.}$;
- Use a smoothed version of the digital elevation model (by means of a blurring kernel) to compute the aspect and slope quantities, yielding a quantity we term $W'_0$;
- Apply empirical adjustments $f_i$ as multiplying factors on top of a non-dimensional $W'_0$ quantity to determine the dimensional vertical velocity $W_{0i}$.

**Fig. 7:**
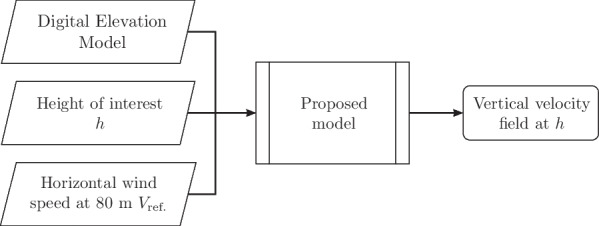
Inputs and outputs for the proposed model. Details regarding the methods behind the *proposed model* block are given in “Model development” section

A summary of inputs and outputs of the model is given in Fig. 7.

### Model validation with field observations

As discussed in the previous sections, the model was developed from LES results, and while the SOWFA code used in this work has also been used and validated in the wind energy and atmospheric modeling communities (see references given in “Large-eddy simulation methodology” section), vertical velocity is not a quantity often investigated in detail. Therefore we sought to compare the model results with field data. Our particular interest is in measured vertical velocities at heights spanning a few hundred meters AGL, corresponding to the rotor-swept zone of typical land-based wind turbines.

Several candidate field studies were considered, including Bolund hill, Askervein hill, Perdigao mountain, and Alaiz mountain. Bolund hill is a small peninsular feature that is 12 m high, 130 m long, and 75 m wide located near the city of Roskilde in Denmark. Its geometrical shape induces complex three-dimensional flow. The experimental campaign [3], however, contains masts that are only 10 m tall. Askervein hill [60], located in Scotland, has a nearly Gaussian shape with mild slopes. The campaign, performed in 1985, has masts as tall as 50 m, but most of the data are taken using significantly shorter masts. Most importantly, however, no vertical component of the velocity is given, only statistics of such component. Perdigao mountain was the site of a thorough field campaign, conducted in 2018 [18]. Perdigao is a double-ridge case, located in Portugal, selected for its challenging interaction of one ridge’s wake on the other. The campaign had tens of masts spread across the region, and three of them contains vertical velocity data up to 100 m AGL. We looked at several months worth of data and noted very strong diurnal cycle characteristics imprinted on the vertical velocity time-history. That means that the region was subject to strong convective effects during daytime and stable boundary layers at nighttime. Both the model developed in this work and the original one are not designed for non-neutral conditions, so they are not applicable to the conditions observed during the campaign.

**Fig. 8:**
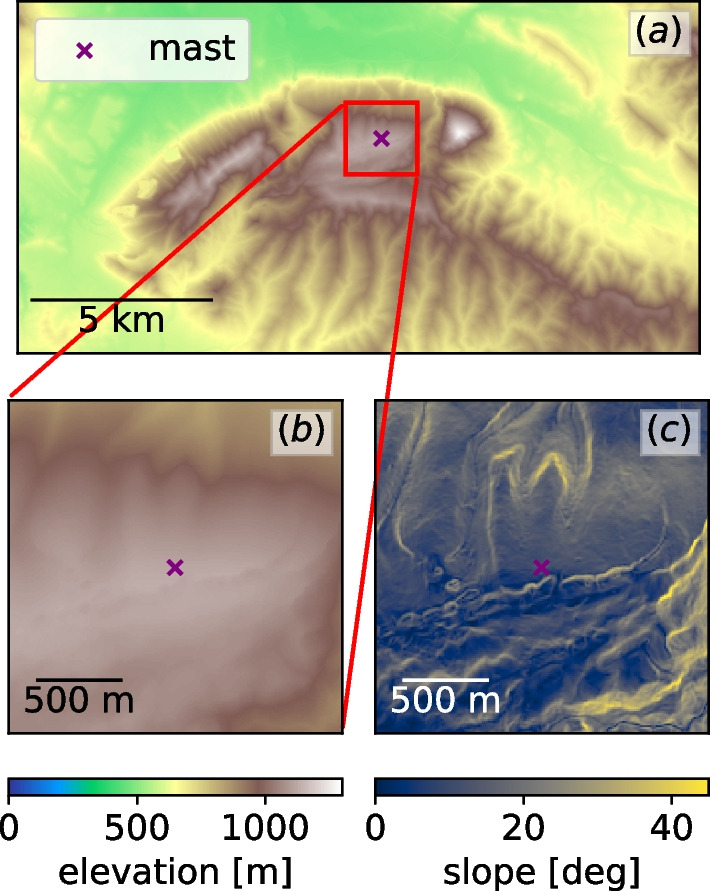
Overview of the Alaiz mountain region. **a** General surroundings with the MP5 mast location shown by a marker. Zoom around the MP5 mast, **b** elevation; **c** slope

For model validation we selected the Alaiz mountain, Spain field campaign. The Alaiz region is presented in Fig. 8. It is a mountain where flows from every direction are subject to complex orographic features. In particular, southerly flows experience complex mountainous regions with small ridges before a steep incline, and northerly flow is subject to a steep incline. A DEM of the Alaiz mountain region with 2-m resolution was provided by authors of the experiment.

We selected hilltop reference mast MP5 that contained instruments and measurements of the vertical velocity at heights up to 118 m AGL was selected (see Fig. 8). Three-dimensional wind speed data collected by sonic anemometers were available between the months of July 2017 and July 2019. We focused on the period spanning July to Dec 2017. The three components of the wind speed are available in 10-min interval means, including the maximum, minimum, and standard deviation of several variables within each 10-min interval. For this validation exercise, we focus on the comparison of vertical velocity component only.

No strong convective updrafts were observed in the data, suggesting the orographic lift conditions for which the model was developed. We assume that any thermally induced vertical disturbances present are averaged out in the 10 min windows. Buoyant disturbances are naturally present in the atmospheric boundary layer due to the heat flux from the ground surface. Given vegetation and other ground irregularities present at the site, localized temperature differences are generally random. Large pockets of positive and negative vertical velocity develop. When coupled with a non-zero horizontal wind speed, the disturbances vary in time and space in an unpredictable manner. Our assumption is rooted in numerical experiments of unstable boundary layers and holds true for canonical unstable simulations with uniform heat flux for averaging periods as short as 5 min. Further analysis of the temporal window of the available data was performed, indicating thermal updrafts had no impact on the data.

Comparison between the field data and both the original and proposed models were done for every 10-min-mean datapoint. For each datapoint, we obtain the wind direction and the horizontal wind direction, which are directly used in both the $\textsc {BO04}$ and the proposed model. Time-series of the vertical velocity is then obtained for both models and simple statistics metrics are obtained. Some times with missing field data indicate that the vertical velocity was not available at those times, but horizontal velocity was, and thus the modeled values could be determined. For details on the instruments and data processing and quality, the reader is referred to the experiment’s original references [37, 52]. Results of the validation with field observations are presented in “Model validation with field data” section.

### Model illustration with movement model simulations

In the final part of the study, our goal was to determine the extent to which simulated movement tracks of a soaring bird are dependent on the updraft model chosen, and whether the proposed updraft model yields more realistic results at higher flight altitudes where orographic lift weakens. We use two sites with different terrains and wind flow regimes and estimate the updraft using the $\textsc {BO04}$ model and the proposed model at two different heights. The flight tracks are simulated with a heuristic individual-based movement model (IBMM) within the Stochastic Soaring Raptor Simulator framework [50].

In the IBMM, the simulated bird reduces its energy expenditure by selecting a movement path based solely on local updraft availability and intensity above a threshold value. This is a purposely simplistic approach that does not consider the possibility that the bird makes movement decisions based on the overall structure of the landscape/updraft field within view; rather, it assumes that the bird responds rapidly to updraft conditions experienced during flight in its immediate vicinity [32]. The model has only two parameters: a specified direction of motion ($\phi$) and the species-dependent threshold updraft velocity ($w_\text {thr}$) that will sustain soaring/gliding flight. In this example we use 0.85 m/s for $w_\text {thr}$, based on a minimum sink speed calculation [43] using data from Lish et al. [35] for golden eagles. The model track simulation goes as follows: the individual moves in constant-length steps (here, we use 30 m) across the domain from a specified starting location. At each step it assesses updraft availability and intensity along a 60 degree arc one step ahead of its current position (at $\phi$, $\phi \pm 15$ deg, $\phi \pm 30$ deg). The modeled updraft velocity field is bilinearly interpolated to these locations. If the interpolated updraft velocity at one or more of these five locations exceeds $w_\text {thr}$, the individual moves to the location of maximum updraft. If not, it selects its next location randomly (i.e., the movement path becomes a directed random walk). The model is run for 1000 simulated tracks across the model domain from various starting positions along a model boundary, and a presence map is generated using a Gaussian smoothing filter over the 1000 tracks.

**Fig. 9:**
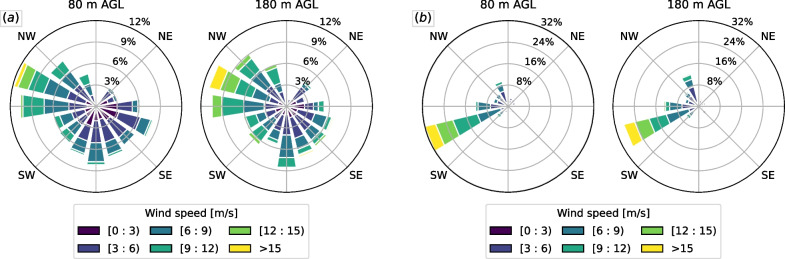
Wind roses for daytime hours at two different heights for **a** fall season at the Appalachian mountains in central Pennsylvania; **b** spring season at the Altamont Pass region in California

The two sites selected in this example are in central Pennsylvania within the Valley and Ridge physiography of the Appalachian Mountains, and near Altamont Pass in the Diablo Range of California. The central Pennsylvania site, a different site than one previously used to develop the model, is dominated topographically by distinct and steep northeast-to-southwest trending ridges, whereas the Altamont Pass region is less well-structured, with a mix of mildly sloping and steeper terrain, including many small-scale features that modify the windfield. Using the WIND Toolkit, mean wind conditions are obtained for the fall season (September–November) for the Appalachian Mountains region, and for the spring season (March–May) for the Altamont Pass region. We obtain the mean wind conditions at two different heights: 80 m and 180 m above ground level; 80 m is the reference height of the proposed model, and 180 m is the highest height where atmospheric data are available through the WIND Toolkit. The seasons were selected because they represent migratory seasons with significant movement activity in each respective region. An analysis of WIND Toolkit wind roses during daytime hours for each season was performed with the goal of selecting both typical and atypical (although realistic) conditions for each site. Wind roses for each site at each height are shown in Fig. 9.

A single wind direction was selected for each site, corresponding to the prevailing west-northwest direction for the Appalachian region, and west-southwest for the Altamont Pass region. For both locations we obtained the mean and the standard deviation of the wind speed at the prevailing wind direction. We devised three wind conditions, based on the mean value plus or minus one standard deviation. We refer to these synthetic conditions as “low,” “moderate,” and “high” wind conditions, for each height and for each location. The final values for each condition are shown in Table 1. The updrafts were computed for each wind condition from Table 1 using both $\textsc {BO04}$ and the proposed model. The IBMM was run for each case, with the updraft model ($\textsc {BO04}$ vs. proposed) as the only difference between the model runs. Results are presented in “Movement model simulations” section.

**Table 1:** Typical wind conditions for each site used for the movement model simulation

Wind condition	Appalachian		Altamont Pass	
	80 m AGL	180 m AGL	80 m AGL	180 m AGL
Low	5.10 m/s	5.95 m/s	6.60 m/s	6.08 m/s
Moderate	8.36 m/s	9.74 m/s	10.57 m/s	10.32 m/s
High	11.63 m/s	13.52 m/s	14.53 m/s	14.55 m/s

## Results

### Model comparison to LES using real terrains

**Fig. 10:**
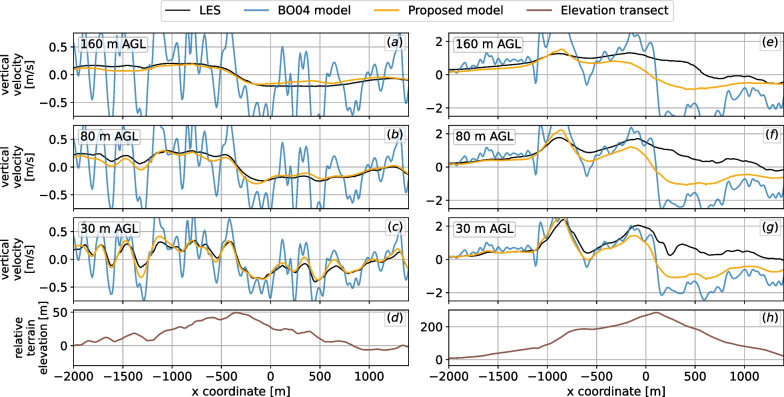
Transects comparing original $\textsc {BO04}$ and improved models to LES considering 8 m/s westerly winds (from left to right). Three heights AGL are shown, as is the terrain transect in the bottom panels. **a**–**d** Wyoming region; **e**–**h** Pennsylvania region

**Fig. 11:**
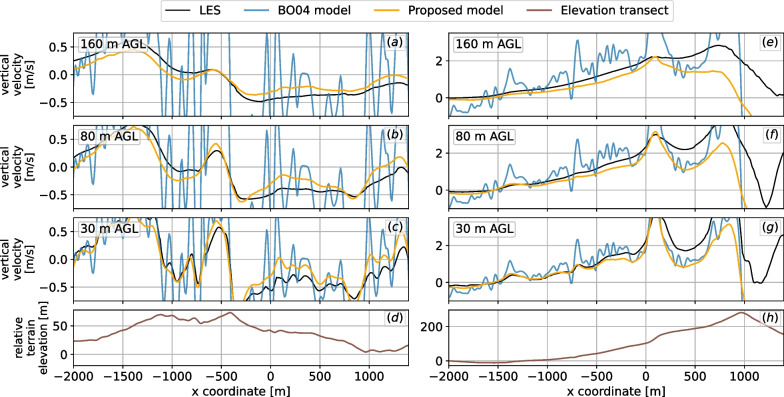
Transects comparing original and improved models to LES considering 15 m/s westerly winds (from left to right). Three heights AGL shown, as well as the terrain transect in the bottom panels. **a**–**d** Wyoming region; **e**–**h** Pennsylvania region. The strong winds induce very high values of updrafts near steep slopes

A comparison of both the original $\textsc {BO04}$ and the proposed model against the LES results is shown in Fig. 10 for the 8 m/s wind speed case and in Fig. 11 for the 15 m/s case. In order to better visualize the differences, we present transects of the terrain. In the 8 m/s case, the flow over the Wyoming region follows the underlying geometry without separation, whereas in the Pennsylvania region, some signs of flow separation and turbulence can be seen around $x=200$ at 30 m AGL. The proposed model follows the LES results quite well but fails to match the highest updrafts observed in the Pennsylvania region. However, the proposed model eliminates the high local variability in vertical velocities that is present in the original $\textsc {BO04}$ model due to local variation in terrain slope. The local variability increases significantly as wind speed increases. Also, as altitude increases, $\textsc {BO04}$ consistently overpredicts vertical velocities, and at 160 m AGL, by as much as 5 times. As noted previously, positive updrafts due to turbulent effects on the leeward side of the terrain are not captured by either model.

Model comparison statistics relative to LES for both the Wyoming and Pennsylvania region for both wind speeds are given in Table 2. The proposed model performs well for all scenarios for the Wyoming site, and for typical wind speeds of 8 m/s for the Pennsylvania site. Overall, it shows improved performance over $\textsc {BO04}$ (relative to LES) for 10 of the 12 scenarios investigated. As expected the $\textsc {BO04}$ model performance relative to LES degrades as altitude increases as it is a two-dimensional model. The proposed model bias relative to LES varies from slightly positive for the Wyoming site to negative for the Pennsylvania site. $\textsc {BO04}$ consistently shows strong positive bias. Model performance relative to LES declines for steep geometries (Pennsylvania case) under high wind conditions due to increasing turbulent effects.

**Table 2:** Statistics of the difference of the vertical velocity obtained using LES and simplified models for the Wyoming and Pennsylvania regions shown in Figs. 10 and 11

Model	Height AGL	Wyoming				Pennsylvania			
		8 m/s		15 m/s		8 m/s		15 m/s	
		*μ*	*σ*	*μ*	*σ*	*μ*	*σ*	*μ*	*σ*
BO04	30 m	0.17	0.20	0.31	0.36	0.04	0.44	0.00	0.93
Proposed		0.03	0.08	0.05	0.15	− 0.09	0.37	− 0.22	0.79
BO04	80 m	0.28	0.28	0.53	0.53	0.23	0.52	0.37	0.99
Proposed		0.01	0.07	0.03	0.13	− 0.11	0.32	− 0.25	0.67
BO04	160 m	0.37	0.33	0.70	0.66	0.48	0.68	0.85	1.26
Proposed		− 0.02	0.05	0.01	0.10	− 0.17	0.28	− 0.36	0.57

For the statistics presented, only regions of modeled positive vertical velocity are considered. Mean $\mu$ and standard deviation $\sigma$ given in units of m/s

### Model validation with field data

**Fig. 12:**
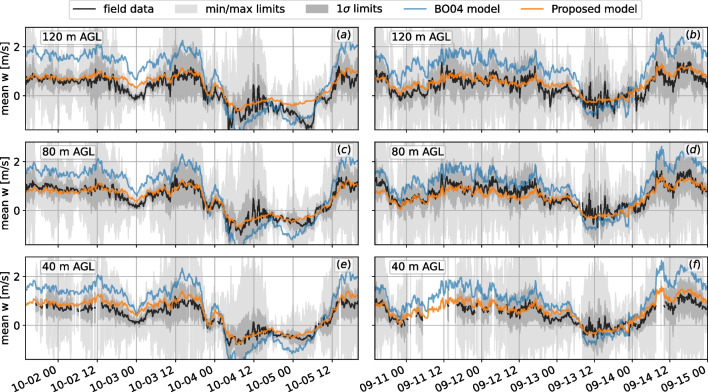
Time history of vertical velocity at three heights at the MP5 mast at Alaiz mountain. Field data shown are 10-min averages and corresponding statistics of each interval and are shown alongside $\textsc {BO04}$ and proposed model estimations. **a**, **b** 118 m AGL; **c**, **d** 80 m AGL; **e**, **f** 40 m AGL

Sample 2-day time series of vertical velocity for hilltop mast MP5 at the Alaiz site are shown in Fig. 12 along with the results for both models. The mean vertical velocity *w* component for each 10-min interval is presented, as are the minimum, maximum, and limits of one standard deviation. At the MP5 location, the flow characteristics depend on the wind direction, and thus, for every snapshot in time, we determined the updraft value using the mean wind direction at the interval.

**Fig. 13:**
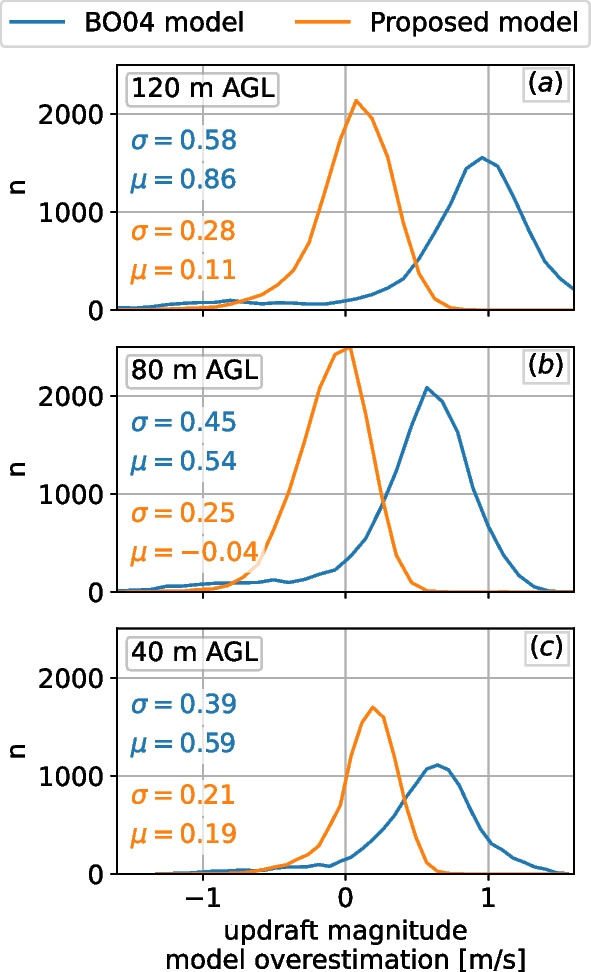
Histogram of the differences between observed and estimated vertical velocity at the MP5 mast at Alaiz mountain, July–December 2017. A positive value represents an overestimation by the model. The values shown in the plots are the mean and standard deviation in m/s: **a** 118 m AGL; **b** 80 m AGL; **c** 40 m AGL

We calculated differences between the observed values and the model estimates for the July–December 2017 data. The differences for the positive vertical velocities (updrafts) are shown in terms of model overestimation in histogram form in Fig. 13. A positive value means the model overestimated the updraft value as compared to field data, whereas a negative value means the model underestimated it. The histogram of the differences in updraft estimations reveals a slight bias of the proposed model to overpredict the updraft magnitude, whereas the $\textsc {BO04}$ model bias is significantly stronger. At 120 m elevation, the proposed model reduces the mean error from 0.86 to 0.11 m/s ($\sigma$ from 0.58 to 0.28 m/s), a mean improvement of 87%. At 80 m AGL, the mean error went from 0.54 to 0.04 m/s ($\sigma$ from 0.45 to 0.25 m/s), a mean improvement of 92%. Finally, closer to the ground at 40 m, the improved model reduced the mean error on the updrafts from 0.59 to 0.19 m/s ($\sigma$ 0.39 to 0.21 m/s), a reduction of 68%. The least improved height was 40 m AGL, which is expected because this height was at the edge of the suite of heights in the parametric study and was therefore more sensitive to the curve-fit performed.

### Movement model simulations

**Fig. 14:**
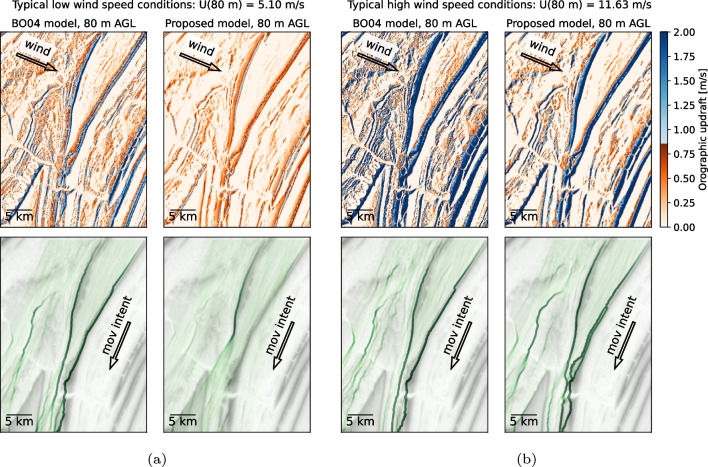
Orographic updrafts resulting from wind at the direction indicated (top row), and the resulting presence map (bottom row) for fall migration in the Appalachian region. The updraft color scale is such that it is blue when updrafts are above the 0.85 m/s threshold and can be used for orographic soaring. The bottom row includes the elevation map in light gray combined with relative presence density in green (darker green indicates higher density). The starting position of the simulated birds is near the north boundary, and their movement intention is shown by the arrow. **a** Typical low wind speed conditions at 80 m AGL; **b** typical high wind speed conditions at 80 m AGL

**Fig. 15:**
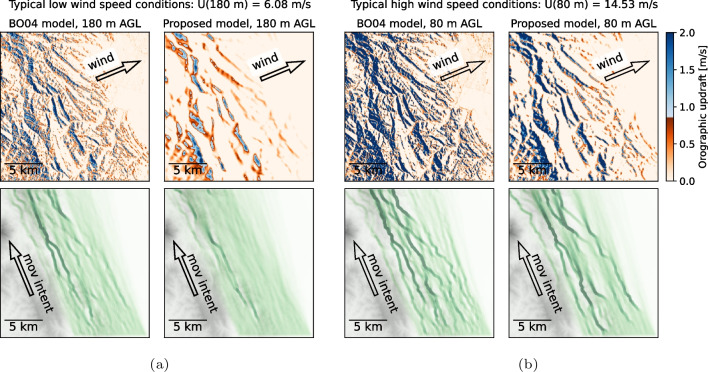
Orographic updrafts and simulation model results for the Altamont Pass region for northward (spring) movement starting along the southern boundary. **a** Typical low wind speed conditions at 180 m AGL; **b** typical high wind speed conditions at 80 m AGL

In this section we examine the impact of the choice of orographic updraft model on tracks of simulated eagles. Selected results of flight tracks and presence maps simulated with the IBMM are shown in Figs. 14 and 15.

First, for the Appalachian region, results for 80 m AGL at low and high wind speed conditions are shown in Fig. 14. For the low-speed case, the updrafts are very different—the original model shows large regions where the updraft is above the threshold value of 0.85 m/s, indicated by the blue colormap, whereas the proposed model is more conservative and only one ridge distinctively has updrafts above the threshold. We simulate 1000 virtual golden eagles; their initial position is along the north boundary, and their direction intent $\phi$ is SSW (indicated by the arrow in the figure). The resulting presence map is computed by showing all the tracks superimposed and applying a blurring kernel. For a scenario where there is no preferential path, all the simulated eagles take a random walk approach and the resulting presence map appears like a uniform light shade of color. In a scenario where the preferential paths are similar between both orographic updraft models, we expect the presence map to be similar. In Fig. 14a most of the area has below-threshold updrafts by the proposed model, which results in most of the domain showing a directed random walk behavior, except for the ridge around the center of the domain, where a certain amount of updraft above the threshold weights in the heuristics rules to attract the bird to that area. The original model, on the other hand, has many regions with updrafts above the threshold, and thus the simulated presence maps have many well-defined preferential paths. We note that at 180 m AGL and low wind speed (not shown here), there is effectively no orographic updraft above the threshold for the proposed model, and the resulting presence maps become directed random walks, as expected. On the other extreme, where above-threshold updrafts are widely available, the two models result in similar presence maps. For instance, Fig. 14b shows a high wind condition at 80 m AGL, a situation where both models estimate large updraft magnitudes at the Appalachian region. While their magnitude is lower on the proposed model, the spatial pattern is very similar. The presence maps are similar with the exception of the lower center portion of the domain. Due to the local terrain and the wind direction, the proposed model correctly captures the updrafts caused by two mountain peaks in close proximity. The sheltering angle measure ensures the updrafts are stronger on the upstream ridge, meaning the virtual eagles will prefer that path. On the original $\textsc {BO04}$ model, the birds choose a slightly different path.

For the Altamont Pass region, for every wind condition investigated (Table 1), there are clear differences between the $\textsc {BO04}$ and the proposed model, with a smoother updraft pattern in the proposed model. Figure 15 shows two wind conditions and the differences observed on the presence maps of 1000 virtual eagles with a NNW movement intent. We select the high and low wind speed conditions, highlighting the extremes observed in that area. Even at the reference height of 80 m, the resulting updraft field is smoother, resulting in fewer, more defined pathways with high orographic updraft, as opposed to several small ridges. The original model results in tracks that are more spread across slightly different ridges following the same direction. That is observed in both cases shown in Fig. 15 for the $\textsc {BO04}$ model. For the low wind speed case, Fig. 15a, the proposed model yields few preferred movement paths and gives random walks over most of the domain, a reflection of estimated updraft velocities being below the threshold for a large portion of the migration path. For the high wind speed case, both models result in several areas of usable, above-threshold updraft. The difference in Fig. 15b is that the proposed model smooths the field, resulting in fewer well-defined tracks.

## Discussion

In this work we propose a new model for rapid estimation of the vertical component of wind over complex terrain. The primary application is in understanding, modeling, and predicting the movements of soaring birds using orographic updrafts at altitudes within a few hundred meters AGL. The model is based on a set of corrections or adjustments applied to the $\textsc {BO04}$ model.

The corrections to $\textsc {BO04}$ were developed based on LES numerical simulations of canonical neutrally stratified flows subject to interactions with complex terrain. A parametric study was performed on synthetic Gaussian hills to understand the effects of isolated orographic features on the vertical velocity field, and further adjustments were made using LES modeling of flow over real terrain in two distinct physiographic regions, representative of varying terrain complexity. One of the adjustments extends the model to three dimensions by explicitly accounting for the height AGL (Eq. (2)). Another adjustment takes into consideration wind conditions at a reference height, which can be obtained from numerical weather prediction models such as the WIND Toolkit [16], maintained by the National Renewable Energy Laboratory, or the High-Resolution Rapid Refresh [15] model, maintained by the National Oceanic and Atmospheric Administration. Here, we have used 80 m as the reference height, consistent with the typical hub height of land-based wind turbines, and the availability of data from both field experiments and analysis data sets. Another adjustment considers orographic features upstream of the point of interest in order to properly account for larger-scale flow effects, such as exposed, channeled, or sheltered areas of flow (Eqs. (3–5)). A final adjustment includes a measure of terrain complexity, allowing the model to be generalized to regions that include different terrain features (Eqs. (7–8)).

By comparison to numerical results using the validated SOWFA LES model, we show that the revised model produces a smoother and more realistic updraft field than $\textsc {BO04}$, much less dependent on variation in the *local* terrain slope of the DEM. This is clearly illustrated in Figs. 10 and 11 for two distinct terrains. Additionally, the revised model more accurately represents the lateral extent of the updraft field upwind of obstacles, by explicitly including the macroscale effects of terrain features on the flow pattern in the model formulation.

Model validation was performed with 10-min windows of wind data collected at three different elevations at the Alaiz site in a region of complex terrain. On average, using 5 months of collected data, the proposed model predictions were improved over $\textsc {BO04}$ by as much as 94% at a typical hub height of land-based wind turbines (80 m), reducing the standard deviation from 0.45 to 0.25 m/s. At the worst-performing elevation, the proposed model improved the updraft mean prediction by 68% over $\textsc {BO04}$. The proposed model overall predicts smoother updraft velocity fields consistent with those observed in the experiment. We note that the mean bias present in the original $\textsc {BO04}$ model for this data is in some cases of the same order of magnitude as the updraft threshold for soaring birds (e.g., 0.85 m/s for golden eagles as mentioned earlier). In a movement modeling context, the practical implication is that there is a strong potential for overestimation of regions of usable, updraft for soaring flight when using $\textsc {BO04}$. Finally, we showed examples of how the updraft model choice may impact predicted golden eagle flight paths using a simple IBMM in which movement choices are based on local orographic updrafts.

Based on these results, we suggest that $\textsc {BO04}$ only be used in cases of large-scale movement patterns where fine-scale details (less than hundreds of meters) and the vertical structure of terrain updrafts are not important to the objectives of the study. Of particular relevance are cases in which, due to low slope angles or wind speeds, the vertical velocity ($w_{0_i}$) is near in magnitude to the updraft threshold ($w_\text {thr}$) needed to support soaring flight. In “Movement model simulations” section, using an updraft threshold ($w_\text {thr}$) of 0.85 m/s for golden eagles, we found significant differences in simulated movement patterns (see Figs. 14a and 15a) under low wind conditions, with fewer concentrated flightlines using the proposed model. In other cases with stronger winds and updrafts well in excess of $w_\text {thr}$ (see Figs. 14b and 15b), the differences between the results are minor. This demonstrates that the $\textsc {BO04}$ model should be applied with care. In applications where detailed three-dimensional movement patterns are being analyzed or modeled, such as in understanding flight behavior near wind turbines or in developing new tools for micro-siting turbines on the landscape, the revised model will provide much more reliable estimates than with $\textsc {BO04}$.

We caution that despite these improvements, the revised model has limitations. The model is empirical, and although it gives good results for the cases described, additional improvements and/or tuning may be needed to handle more complex and steep terrains. In particular, it is noted that the model is not applicable downwind of terrain where the vertical velocities are negative, nor to combinations of wind speeds and slopes that will induce strong separation and create recirculation zones downwind of terrain. The flow within these zones is highly complex, and the overall mean vertical velocity value is more dependent on turbulent characteristics of the flow than on the underlying terrain. We also highlight that the model should be applied in the altitude range of a few hundred meters above the ground. In addition, we note that at higher elevations, the influence of the underlying terrain on the wind becomes increasingly small in regions of moderately complex terrain. Finally, although our focus in this work was on movement ecology applications, another area that can benefit from the model proposed in this work is that of autonomous, high-endurance soaring sailplanes, where an accurate prediction of available orographic updrafts above a particular threshold can also help inform movement decisions.

## Conclusion

Despite its extensive use in the movement ecology literature, $\textsc {BO04}$ is a highly simplified representation of a complex turbulent process. The model proposed here overcomes several important limitations of $\textsc {BO04}$, providing a more robust method for simulating orographic updrafts in three dimensions over sloping terrain. It provides realistic matches to LES simulations and to field data, and is applicable to flow modeling at scales relevant to movement ecology studies of soaring birds. We note again that the model is available to the public on GitHub, under the project “Engineering Vertical Velocity Estimator” [62]. Resource requirements are low, and the model can be executed on a personal laptop in a matter of a few seconds to few minutes, depending on the spatial domain extent and the number of wind directions desired.

## Data Availability

The code for this work is available at: Engineering Vertical Velocity Estimator (EVVE), www.github.com/nrel/evve [62]. Input files for LES cases executed in this work are available from leading author upon request.
